# Protection by the Total Flavonoids from *Rosa laevigata* Michx Fruit against Lipopolysaccharide-Induced Liver Injury in Mice via Modulation of FXR Signaling

**DOI:** 10.3390/foods7060088

**Published:** 2018-06-08

**Authors:** Lile Dong, Xu Han, Xufeng Tao, Lina Xu, Youwei Xu, Linlin Fang, Lianhong Yin, Yan Qi, Hua Li, Jinyong Peng

**Affiliations:** College of Pharmacy, Dalian Medical University, Western 9 Lvshunnan Road, Dalian 116044, China; gulinadalian2009@163.com (L.D.); Xuhan0118@163.com (X.H.); taoxufengdalian@163.com (X.T.); Linaxu0112@163.com (L.X.); Youweixu0112@163.com (Y.X.); qimengdy2016@163.com (L.F.); Lianhongyin0112@163.com (L.Y.); lihua2014dy@163.com (H.L.)

**Keywords:** *Rosa laevigata* Michx, total flavonoids, lipopolysaccharide, FXR signal, oxidative stress and inflammation, lipid metabolism

## Abstract

We previously reported the effects of the total flavonoids (TFs) from *Rosa laevigata* Michx fruit against carbon tetrachloride-induced liver damage, non-alcoholic fatty liver disease, and liver ischemia-reperfusion injury. However, there have been no papers reporting the role of *R. laevigata* TFs against lipopolysaccharide (LPS)-induced liver injury. In this paper, liver injury in mice was induced by LPS, and *R. Laevigata* extract was intragastrically administered to the mice for 7 days. Biochemical parameters in serum and liver tissue were examined, and pathological changes were observed by transmission electron microscopy, hematoxylin and eosin (H&E) and Oil Red O staining. The results showed that the TFs markedly reduced serum ALT (alanine transferase), AST (aspartate transaminase), TG (total triglyceride), and TC (total cholesterol) levels and relative liver weights and improved liver pathological changes. In addition, the TFs markedly decreased tissue MDA (malondialdehyde) level and increased the levels of SOD (superoxide dismutase) and GSH-Px (glutathione peroxidase). A mechanistic study showed that the TFs significantly increased the expression levels of Nrf2 (nuclear erythroid factor2-related factor 2), HO-1 (heme oxygenase-1), NQO1 (NAD(P)H dehydrogenase (quinone 1), GCLC (glutamate-cysteine ligase catalytic subunit), and GCLM (glutamate-cysteine ligase regulatory subunit) and decreased Keap1 (Kelch-like ECH-associated protein 1) level by activating FXR (farnesoid X receptor) against oxidative stress. Furthermore, the TFs markedly suppressed the nuclear translocation of NF-κB (nuclear factor-kappa B) and subsequently decreased the expression levels of IL (interleukin)-1β, IL-6, HMGB-1 (high -mobility group box 1), and COX-2 (cyclooxygenase-2) by activating FXR and FOXO3a (forkhead box O3) against inflammation. Besides, the TFs obviously reduced the expression levels of SREBP-1c (sterol regulatory element-binding proteins-1c), ACC1 (acetyl-CoA carboxylase-1), FASN (fatty acid synthase), and SCD1 (stearoyl-coenzyme A desaturase 1), and improved CPT1 (carnitine palmitoyltransferase 1) level by activating FXR to regulate lipid metabolism. Our results suggest that TFs exhibited protective effect against LPS-induced liver injury by altering FXR-mediated oxidative stress, inflammation, and lipid metabolism, and should be developed as an effective food and healthcare product for the therapy of liver injury in the future.

## 1. Introduction

Acute liver injury (ALI), with high incidence and mortality, refers to the acute injury of liver cells caused by various factors, which can cause long-term structural damage with progressive fibrosis and repercussions on liver function [1,2,3]. Many factors can cause ALI, among which the chemical named lipopolysaccharides (LPS) that exists in the outer membranes of gram-negative bacteria [4,5]. LPS can cause serious global problems of sepsis and stimulate the immunological system, causing sudden cessation of the normal liver function and severe liver damage [6].

Nowadays, the underlying mechanisms of LPS-induced liver injury are still not well understood. However, several biological processes, including oxidative stress, inflammation, and lipid metabolism have been examined in LPS-induced liver injury [7,8,9]. Farnesoid X receptor (FXR), a ligand-activated transcription factor which belongs to the nuclear hormone receptor superfamily, is highly expressed in hepatic tissue [10]. The activation of hepatic FXR can regulate the expression levels of various genes, including nuclear erythroid factor 2-related factor 2 (Nrf2), forkhead box O3 (FOXO3a), and sterol regulatory element-binding proteins-1c (SREBP-1c) [11,12,13]. Briefly, Nrf2 is present in the cytoplasm and binds to Kelch-like epichlorohydrin (ECH)-associated protein 1 (Keap1) in normal conditions. However, Nrf2 translocates into the nucleus and activates its target genes when FXR triggers the complex Nrf2-Keap1, regulating various anti-oxidative genes, including hemeoxygenase-1 (HO-1), NAD(P)H hdehydrogenase (quinone 1) (NQO1), glutamate-cysteine ligase catalytic subunit (GCLC), and glutamate-cysteine ligaseregulatory subunit (GCLM) against oxidative stress [14]. In addition, FXR can also inhibit the transcriptional activity of nuclear factor kappa B (NF-κB) by stimulating FOXO3a and reduce the levels of pro-inflammatory cytokines, including interleukin-1 beta (IL-1β), interleukin-6 (IL-6), tumor necrosis factor alpha (TNF-α), high-mobility group box 1 (HMGB-1), and cyclooxygenase-2 (COX-2), suppressing inflammation [15]. Besides, FXR can decrease the expression levels of SREBP-1c, fatty acid synthase (FASN), acetyl-CoA carboxylase-1 (ACC1), and stearoyl-coenzyme desaturase-1 (SCD1) and increase carnitine palmitoyl transferase 1 (CPT1) level to regulate lipid metabolism [16]. Therefore, upregulating FXR to suppress oxidative stress, inflammation, and lipid metabolism represents a potential treatment strategy against liver LPS-induced injury. 

*Rosa laevigata* Michx, a member of the *Rosaceae* family, has long been used to treat diarrhea and cure frequent micturition [17]. The total flavonoids (TFs) from it mainly contains flavones and flavonols, including quercetin, kaempferide, apigenin, and isorhamnetin [18,19]. Our previous studies have demonstrated that the extract has hepatoprotective effects against carbon tetrachloride -induced liver damage, non-alcoholic fatty liver disease, and liver ischemia- reperfusion injury [20,21,22]. However, there are no papers reporting the role of *R. laevigata* TFs against LPS-induced liver injury, as far as we know. Therefore, in the present work, we aimed to investigate the effects and possible mechanisms of action of the TFs against LPS-induced liver injury in mice.

## 2. Materials and Methods

### 2.1. Chemicals and Reagents

LPS was purchased from Sigma-Aldrich (St. Louis, MO, USA). Silymarin was purchased from Sigma Chemical Company (Milan, Italy). Detection kits for ALT (Code No. C009-2), AST (Code No. C010-2), SOD (Code No. A001-1), MDA (Code No. A003-1), GSH-Px (Code No. A005), TG (Code No. A110-1), and TC (Code No. A111-1) were all supplied by Nanjing Jiancheng Institute of Biotechnology (Nanjing, China). The Tissue Protein Extraction Kit was produced by KeyGEN Biotech. Co., Ltd. (Nanjing, China). The Enhanced Bicinchoninic Acid (BCA) Protein Assay Kit was purchased from Beyotime Institute of Biotechnology (Haimen, China). Tris (hydroxymethyl) aminomethane (Tris), sodium dodecyl sulfate (SDS), 4’,6’-Diamidino-2-phenylindole (DAPI), and Oil Red O were obtained from Sigma (St. Louis, MO, USA). Hematoxylin (Code No. ZLI9606), eosin (Code No. ZLI9612), and diaminobenzidine (DAB, Code No. ZLI9632) staining kits were purchased from Zhongshan Golden Bridge Biotechnology (Beijing, China). TransZol, TransStart Top Green qPCR SuperMix, and Trans-Script All-in-One First-Strand cDNA Synthesis SuperMix for qPCR (One-Step gDNA Removal) were purchased from Transgen Institute of Biotechnology (Beijing, China).

### 2.2. Herbal Material and Preparation of TFs

The total flavonoids (TFs) were obtained from *R. laevigata* fruits according to our previous reports [20,21]. Briefly, *R. laevigata* Michx fruits were crushed and extracted with 60% aqueous ethanol (1:8, *w*/*v*) two times for 2 h each time, under heat reflux. After condensation at 60 °C, the extracted solution was added to one glass column containing D101 macroporous resin (Chemical Plant of Nankai University, Tianjin, China). After elution with water, the fraction eluted with 40% aqueous ethanol was collected and evaporated at 60 °C to dryness [20,21]. The content of the TFs in the crude extract was determined to be 0.813 g/g by a colorimetric method using UV spectrophotometry (UV-3010, Hitachi, Tokyo, Japan) as described in our previous study [23,24]. The TFs were dissolved in saline and stored at 4 °C for subsequent experiments.

### 2.3. Animals

Male C57BL/6 mice (eight-week-old, 18–22 g) were provided by Liaoning Changsheng Biotechnology Co., Ltd. (Quality certificate number: SCXK (Liao) 2015-0001). The mice were housed under standard laboratory conditions with controlled temperature (21 ± 3 °C), relative humidity (60 ± 5%), 12h light-dark cycles, and free access to standard chow diet and water. After one week of acclimatization, the mice were randomly divided into six groups: control group, LPS model group, LPS + TFs (200 mg/kg) group, LPS + TFs (100 mg/kg) group, LPS + TFs (50 mg/kg) group, and LPS + silymarin (200 mg/kg) group. The mice in the treatment groups were intragastrically administered the TFs and silymarin (dissolved in saline) for 7 consecutive days. Meanwhile, the mice in the control and LPS model groups were administered saline. Liver injury was induced in mice with an intraperitoneally injection of LPS (8 mg/kg) 2 h before the last administration [25]. After seven days, the animals were sacrificed, and the blood and liver tissue were collected for further experiments. All experimental procedures were approved by the Animal Care and Use Committee of Dalian Medical University and performed strictly in compliance with the People’s Republic of China Legislation Regarding the Use and Care of Laboratory Animals.

### 2.4. Determination of Biochemical Parameters in Serum and Liver

The relative liver weight was determined. The levels of ALT, AST, TG, and TC in the serum and the contents of SOD, MDA, and GSH-Px in the liver tissues were assayed according to the manufacturer’s instruction included in the kits.

### 2.5. Hematoxylin and Eosin Staining

The liver tissues were fixed in 10% neutral formalin solution, dehydrated with a sequence of ethanol solutions, and embedded in paraffin. The sections (5 μm-thick) were cut, transferred onto glass slides, and stained with hematoxylin and eosin (H&E). The stained samples were examined using a light microscope (Nikon Eclipse TE2000-U, NIKON, Tokyo, Japan) and photographed at 200× magnification.

### 2.6. Oil Red O Staining

The liver specimens were frozen in a freezer (Thermo Fisher Scientific, Waltham, MA, USA) at −80 °C, sectioned by a cryostat, and stained for lipids using Oil Red Ostaining. Then, the sections were assessed for the degree of hepatic steatosis by comparing the degree of accumulation of red lipid droplets and photographed under a light microscope (Nikon EclipseTE2000-U, NIKON, Tokyo, Japan) at 200× magnification.

### 2.7. Transmission Electron Microscopy (TEM) Assay

Fresh liver tissue (<1 mm^3^) from the control, model, and TFs (200 mg/kg)-treated groups were fixed in 2% glutaraldehyde overnight at 4 °C. The samples were treated according to a previously described method [22]. The samples were washed in 0.1 M sodium cacodylate buffer, fixed in 1% osmium tetroxide for 2 h, and then dehydrated in gradient ethanol solutions. Finally, the samples were observed and photographed with an electron microscope (JEM-2000EX, JEOL, Tokyo, Japan).

### 2.8. Immunohistochemical and Immunofluorescent Assays

For the immunohistochemical assays, the paraffin sections were deparaffinized, rehydrated, and then treated with 0.01 mol/L citrate (pH = 6.0) in a microwave oven for 15 min. The deparaffinized sections were incubated in 3% hydrogen peroxide for 30 min, and normal goat serum was used to block nonspecific protein binding for 30 min. Then, the sections were incubated with rabbit anti-Nrf2 (1:1000, dilution) antibodies in a moist box at 4 °C overnight, followed by incubation in biotin-labeled goat anti-rabbit IgGs and horseradish peroxidase-conjugated streptavidin for 15 min. Eventually, the slides were incubated in DAB solution for 10 min at 37 °C. Images were taken by using a light microscope (Nikon EclipseTE2000-U, NIKON, Tokyo, Japan) at 200× magnification.

For the immunofluorescent assays, the paraffin sections were deparaffinized with xylene (two times, 15 min each) and rehydrated with different concentrations of alcohol (100, 90, 80, 70, and 60%) for 5 min, followed by incubation with normal goat serum for 20 min to block nonspecific protein binding. Then, the sections were incubated in a moist box at 4 °C overnight with rabbit anti-FXR antibodies (1:500, dilution), rabbit anti-FOXO3a antibodies (1:1000, dilution),and rabbit anti-NF-κB antibodies (1:1000, dilution),followed by incubation with FITC-conjugated goat anti-rabbit IgGs for 1h.Eventually, the cell nuclei were stained with DAPI (5 mg/mL). The immunostained samples were analyzed by fluorescence microscopy (Olympus, Tokyo, Japan) at 200× magnification.

### 2.9. Quantitative Real-Time PCR Assay

Total RNA samples were isolated from the liver tissue using RNAiso Plus reagent following the manufacturer’s instructions. Then, total RNA was reverse-transcribed into cDNA by using the TransScript^®^ All-in-One First-Strand cDNA Synthesis SuperMix for qPCR (One-Step gDNA Removal) Kit. The levels of mRNA expression were quantified by real-time PCR with SYBR_Premix Ex Taq™ II (Tli RNaseH Plus) and ABI 7500 Real-Time PCR System (Applied Biosystems, Waltham, MA, USA). The forward (F) and reverse (R) primers used for real-time PCR are listed in Table 1. The Ct values of the target genes were normalized to GAPDH. Finally, the unknown template was calculated through the standard curve for quantitative analysis.

### 2.10. Western Blotting Assay

Total protein samples were obtained from the livers using the Tissue Protein Extraction Kit on the basis of the manufacturer’s instructions, and the protein content was determined using a BCA Protein Assay Kit. The protein samples (7 mg/mL) were denatured by mixing with an equal volume of 2× sample loading buffer and boiling at 100 °C for 5 min. The proteins were subjected to 10% SDS-PAGE (sodium dodecyl sulfate polyacrylamide gel electrophoresis) and transferred onto PVDF (polyvinylidene difluoride) membranes (Millipore, Burlington, MA, USA). After blocking the non-specific binding sites for 3 h with 5% dried skim milk in TTBS(10 mM TBS plus 1.0% Tween 20) at room temperature, the membranes were individually incubated overnight at 4 °C with the primary antibodies (Table 2). The membranes were then incubated with a horseradish peroxidase (HRP)-conjugated secondary antibody for 2 h at room temperature at a 1:2000 dilution. Protein expression was detected by using an enhanced chemiluminescence (ECL) method and a Bio- Spectrum Gel Imaging System (UVP, Upland, CA, USA). The intensity values of the protein levels were normalized to GAPDH expression level.

### 2.11. Statistical Analysis

All data were analyzed by conducting One-Way ANOVA using the statistical software SPSS 20.0 (IBM, Armonk, NY, USA) and expressed as mean ± standard deviation (SD). Tukey in post-hoc multiple comparisons was used, to examine the statistical significance between groups. Statistical significance was set at *p* < 0.05 or *p* < 0.01.

## 3. Results

### 3.1. Effects of TFs on LPS-Induced Liver Injury

**Figure 1:**
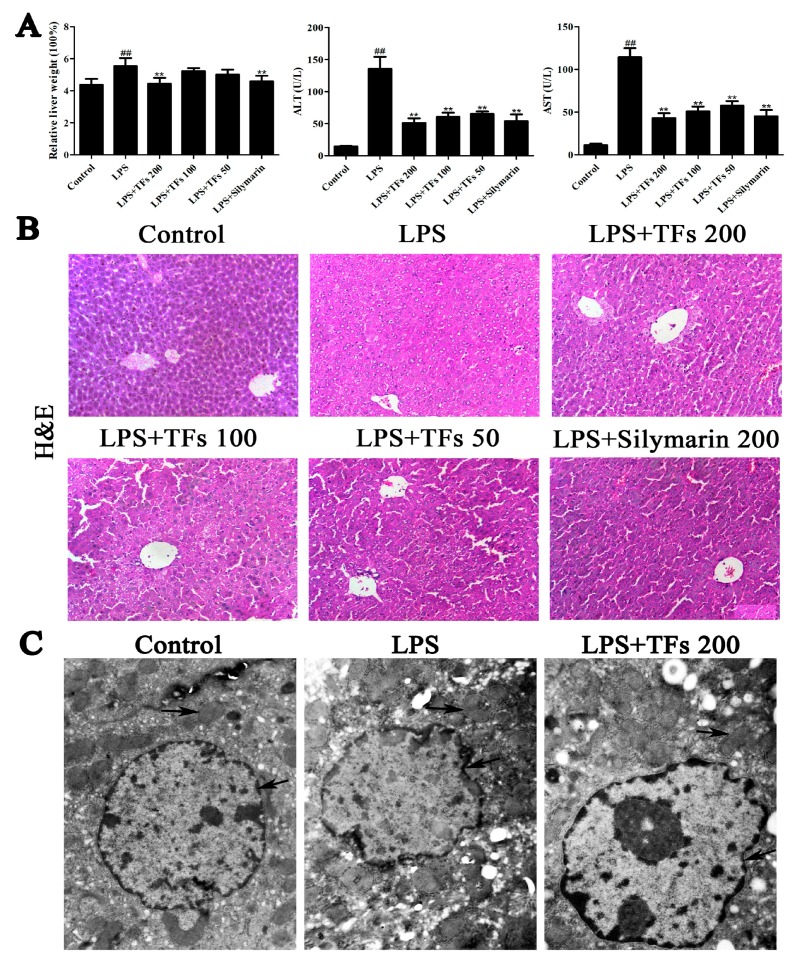
Effects of total flavonoids (TFs) on lipopolysaccharides (LPS)-induced liver injury and cellular structure changes in mice. (**A**) Effects of TFs on relative liver weight, serum ALT (alanine transferase) and AST (aspartate transaminase) activities. (**B**) Hematoxylin and Eosin (H&E) staining of liver tissues (200× magnification,). (**C**) TEM (transmission electron microscope) observations of the nuclei and mitochondria in liver cells (20,000× magnification,). The values are expressed as the mean ± SD (standard deviation) (*n* = 8); ^##^ *p* < 0.01 versus control group; ** *p* < 0.01 versus model group.

As shown in Figure 1A, compared with the control group, the relative liver weight was significantly increased in the model group and was obviously decreased by TFs (*p* < 0.01). The activities of serum ALT and AST in the model group were significantly elevated compared with the control group and were significantly decreased by TFs. As shown in Figure 1B, the results of H&E staining indicated normal liver lobular architecture and cell structure in the control group, while apparent injuries, including large areas of extensive cell necrosis with loss of hepatic architecture and massive inflammatory cells infiltration, were found in the model group and were obviously reversed by TFs.

### 3.2. TFs Improve LPS-Induced Cellular Structure Changes in Mice

As shown in Figure 1C, compared with the control group, severe loss of the structural integrity of the hepatocytes’ nuclei, condensed nuclei, mitochondria valleydis appearance, and mitochondria swelling were clearly found in the model group and were all markedly reversed by the TFs.

### 3.3. TFs Suppresse LPS-Induced Oxidative Liver Injury

**Figure 2:**
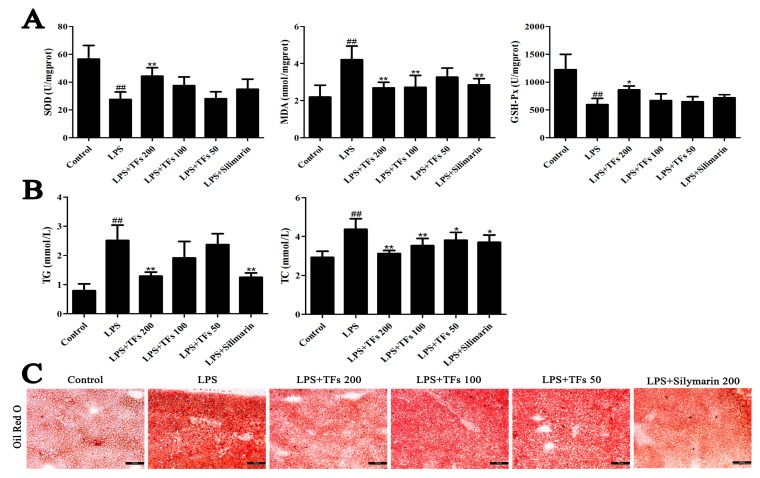
Effects of TFs on LPS-induced oxidative stress and lipid metabolism in mice. (**A**) Effectsof TFs on SOD (superoxide dismutase), MDA (malondialdehyde), and GSH-Px (glutathione peroxidase) levels in the livers of control mice and mice undergoing different treatments. (**B**) Effects of TFs on serum TG (total triglyceride) and TC (total cholesterol) levels in mice. (**C**) Oil Red O staining of liver tissues (200× magnification). The values are expressed as the mean ± SD (*n* = 8); ^##^ *p* < 0.01 versus control group; * *p* < 0.05and ** *p* < 0.01 versus model group.

The antioxidant activities of TFs are shown in Figure 2A. The levels of SOD and GSH-Px in livers were significantly reduced, and the MDA level was significantly increased in LPS-treated mice compared with the control group (*p* < 0.01) and were markedly restored by the TFs.

### 3.4. Effects of TFs on Lipid Metabolism 

The levels of blood lipids are shownin Figure 2B. In the model group, the concentrations of TC and TG in blood were significantly increased, and the amounts of lipid droplets (red) with hepatic neutral lipid accumulation in the liver were significantly increased (Figure 2C),whereas they were decreased by the extract. The data indicated that the TFs had an obvious lipid-lowering effect against LPS-induced liver injury. 

### 3.5. TFs Activate the FXR Signal Pathway

**Figure 3:**
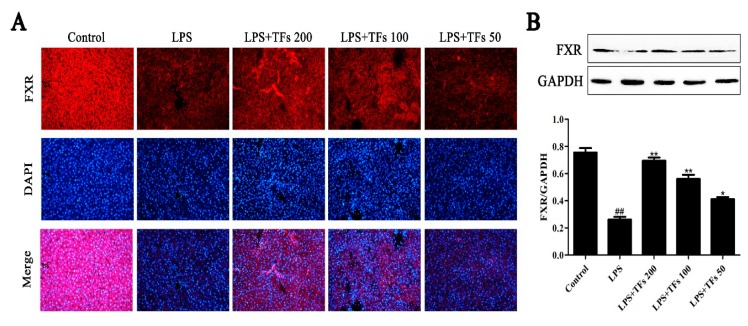
TFs activated the FXR signal pathway. (**A**) Effects of TFs on the expression level of FXR on the basis of the immunofluorescence assay (200× magnification). (**B**) Effects of TFs on the expression level of FXR in liver tissue on the basis of the western blotting assay. The values are expressed as the mean ± SD (*n* = 3); ^##^ *p* < 0.01 versus control group; * *p* < 0.05and ** *p* < 0.01 versus model group. DAPI: 4’,6’-Diamidino-2-phenylindole; FXR: farnesoid X receptor.

The results of the immunofluorescence staining and western blotting assays are shown in Figure 3A,B. Compared with the control group, the expression level of FXR was markedly decreased by LPS in the model group and was significantly upregulated by TFs. These data indicated that TFs attenuated LPS-induced liver injury by upregulatingt he FXR signal pathway.

### 3.6. TFs Regulate FXR-Nrf2-Mediated Oxidative Stress

**Figure 4:**
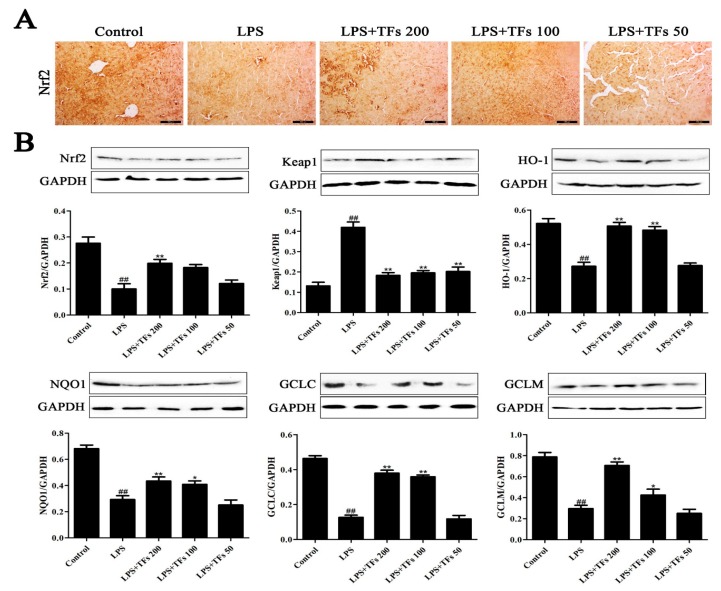
TFs regulated FXR-Nrf2-mediated oxidative stress. (**A**) Effects of TFs on the expression level of Nrf2 in the liver on the basis of the immunohistochemistry assay. (**B**) Effects of TFs on the expression levels of Nrf2 (nuclear erythroid factor 2-related factor 2), Keap1 (Kelch-like ECH- associated protein 1), HO-1 (heme oxygenase-1), NQO1 (NAD(P)H dehydrogenase (quinone 1)), GCLC (glutamate-cysteine ligase catalytic subunit), and GCLM (glutamate-cysteine ligase regulatory subunit) in the liver on the basis of the western blotting assay. The values are expressed as the mean ± SD (*n* = 3); ^##^ *p* < 0.01 versus control group; * *p* < 0.05 and ** *p* < 0.01 versus model group.

As shown in Figure 4A, compared with the model group, more Nrf2-positive areas (brown areas) were found in the control group based in the immunohistochemistry assay, which were increased by TFs. The same results were also verified by western blotting (Figure 4B). Besides, TFs significantly upregulated Nrf2 levels and downregulated Keap1 levels. In addition, the expression levels of downstream proteins, including HO-1, NQO1, GCLC, and GCLM in the model group were significantly decreased, whereas they were significantly increased by TFs. These data suggested that TFs regulated the FXR–Nrf2 signal pathway to suppress oxidative stress.

### 3.7. TFs Regulate FXR-FOXO3a-Mediated Inflammation

**Figure 5:**
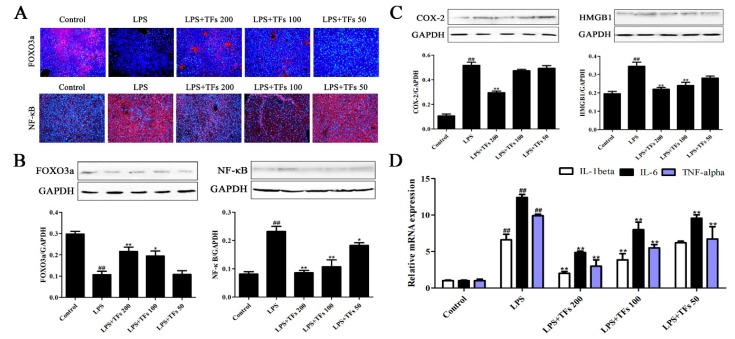
TFs regulated FXR-FOXO3a-mediated inflammation. (**A**) Effects of TFs on the expression levels of FOXO3a and NF-κB in the liver on the basis of the immunofluorescence assay. (**B**) Effects of TFs on the expression levels of FOXO3a and NF-κB in the liver on the basis of the western blotting assay. (**C**) Effects of TFs on the expression levels of COX-2 (cyclooxygenase-2) and HMGB1 (high- mobility group box 1) on the basis of the western blotting assay. (**D**) Effects of TFs on the mRNA levels of IL-1β, IL-6, and TNF-αon the basis of the real-time PCR assay. The values are expressed as the mean ± SD (*n* = 3); ^##^ *p* < 0.01 versus control group; * *p* < 0.05 and ** *p* < 0.01 versus model group.

The immunofluorescence staining and western blotting results of FOXO3a and NF-κB are shown in Figure 5A,B. The expression level of FOXO3a in the model group was increased after LPS induction, and this increase was significantly reversed by TFs. Besides, the protein levels of COX-2 and HMGB1 were notably upregulated (Figure 5C), whereas TFs obviously downregulated their expression levels. Furthermore, the gene expression levels of IL-1β, IL-6, and TNF-α (Figure 5D) were upregulated by LPS and were significantly reduced by the extract. These results suggested that TFs attenuated LPS-induced inflammation by regulating the FXR-FOXO3a signal pathway.

### 3.8. TFs Regulats FXR-SREBP-1c-Mediated Lipid Metabolism

**Figure 6:**
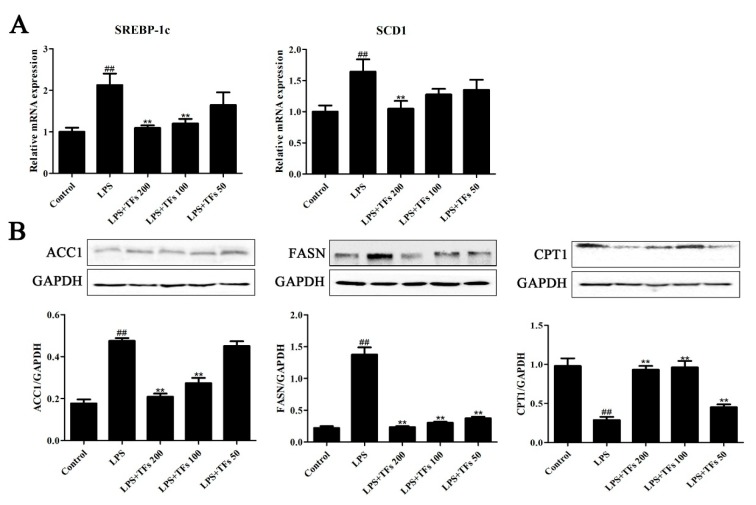
TFs regulated FXR-SREBP-1c-mediated lipid metabolism. (**A**) Effects of TFs on the mRNA levels of SREBP-1c and SCD1 on the basis of real-time PCR assay. (**B**) Effects of TFs on the expression levels ofACC1 (acetyl-CoA carboxylase-1), FASN (fatty acid synthase), and CPT1 (carnitine palmitoyltransferase 1) on the basis of the western blotting assay. The values are expressed as the mean ± SD (*n* = 3); ^##^ *p* < 0.01 versus control group; ** *p* < 0.01 versus model.

As shown in Figure 6A, TFs administration notably reduced the levels of SREBP-1c and SCD1 compared with the model group (*p* < 0.01). In Figure 6B, the protein levels of ACC1 and FASN in model mice were obviously upregulated, while the expression level of CPT1 was significantly decreased. TFs administration (200 mg/kg) markedly reversed their expression levels (*p* < 0.01). These data indicated that TFs regulated LPS-induced lipid metabolism by modulating FXR/SREBP- 1c signaling.

## 4. Discussion

ALI with high degree of morbidity and mortality can cause permanent structural damage, progressive fibrosis, and long-term repercussions on liver function. LPS-induced hepatotoxicity remains a leading cause of death [1,26,27,28]. Currently, no effective therapeutic agents or methods are available for the treatment of ALI. In this study, TFs exhibited significant effects against LPS- induced ALI, as evidenced by the improvement of the decreased AST and ALT levels and by histopathological changes. It has been widely reported that flavonoids from natural foods have favorable antioxidant effects, which can support the pharmacological activities observed in our study of TFs from *R. laevigata* Michx fruits [29,30,31]. Besides, silymarin as a listed liver protecting drug was used as an effective drug in this experiment, and the hepatoprotective effect produced by TFs at the dose of 200 mg/kg was similar to that of silymarin at the same dose.

The mechanisms responsible for LPS-induced ALI are complicated, and previous studies have shown that oxidative stress, inflammation, and lipid metabolism are implicated in LPS-induced liver injury [32,33,34,35]. In terms of oxidative stress, MDA (anindicator of reactive oxygen species (ROS), SOD (an enzyme that can catalyze hydrogen peroxide reaction), and GSH-Px (an enzyme that can catalyze the reduction of hydrogen peroxide and other peroxides) were all markedly improved, and the release of ROS was reduced after TFs pretreatment [36]. In terms of inflammation, NF-κB plays a significant role in regulating the inflammatory response, and activating NF-κB can cause the expression of proinflammatory cytokines, including IL-1β, IL-6, TNF-α, COX-2, and HMGB1 [37]. These proinflammatory cytokines were all reversed by TFs. With respect to lipid metabolism, TFs significantly decreased the levels of TC and TG (indicators of blood lipids) with remarkable lipid-lowering effects [38].

**Figure 7:**
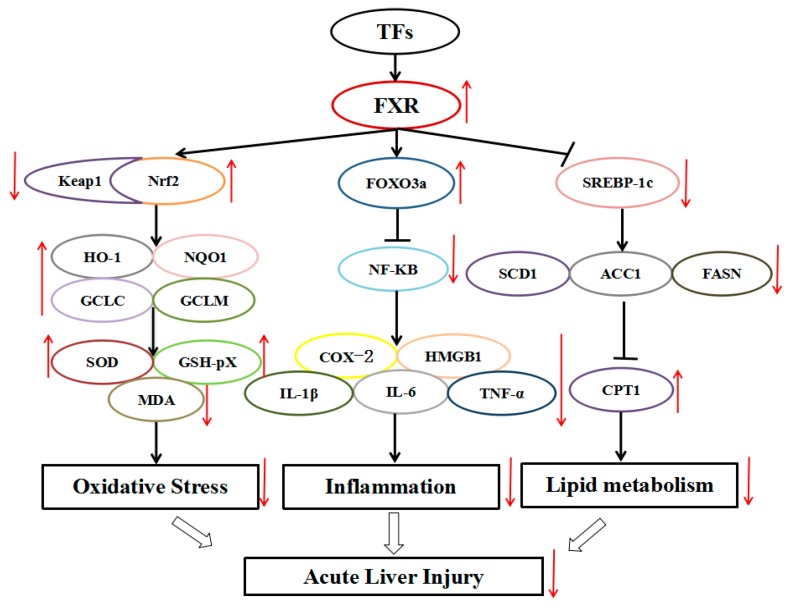
Schematic diagram showing the mechanism of action of TFs against LPS-induced acute liver injury. TFs reduced oxidative stress, inflammation, and lipid metabolism by modulating the FXR signal pathway.

FXR is a member of the nuclear hormone receptor super family, and it was demonstrated that FXR can regulate oxidative stress, inflammation, and lipid metabolism thus improving liver injuries [10,39,40,41]. In our study, TFs exhibited a protective effect against LPS-induced liver injury by enhancing FXR effects on oxidative stress, inflammation, and lipid metabolism (Figure 7).

Nrf2 can regulate intracellular redox homeostasis. It can translocate from the cytosol to the nucleus by changing Keap1 conformation and binding to the antioxidative response elements (ARE) to regulate various anti-oxidative genes, including HO-1, NQO1, GCLC, and GCLM [42,43]. Thus, Nrf2 plays an important role in inhibiting cellular oxidative stress, improving the activities of antioxidant enzymes and reducing ROS-induced damage [44]. In the present work, we believe that the protective effect of TFs against liver injury occurred via the regulation of FXR-Nrf2 signal to suppress oxidative stress.

FOXO3a plays an important role in lymphatic homeostasis and inflammation. Besides, FOXO3a regulates helper T cell activation and tolerance by inhibiting NF-κB activity to modulate inflammation [45]. In the present work, the protective effect of TFs against liver injury was via the regulation FXR-FOXO3a signal to reduce inflammation.

SREBP-1c, a key transcriptional regulator, can increase the expression of key enzymes involved in lipogenesis. FXR can cause the reduction of SREBP-1c mRNA expression and decrease the expression levels of ACC1 and FASN. ACC1 and FASN are two key enzymes involved in lipogenesis, helping acetyl-CoA to form palmitic acid desaturated by SCD1. Besides, the decreased ACC1 levels can increase CPT1 expression, adjusting lipid metabolism [46,47,48]. Therefore, our present data indicated that the protective effect of TFs against liver injury occurred via the modulation of FXR-SREBP-1c signal to adjust lipid metabolism. 

These findings reveal novel mechanisms of action of TFs against LPS-induced acute liver injury, which include the activation of FXR signaling, and suggest that TFs should be developed as an effective food and healthcare product for the therapy of liver injury in the future.

## 5. Conclusions

In summary, our results demonstrate that TFs exhibited a potent effect against LPS-induced liver injury by adjusting FXR-mediated oxidative stress, inflammation, and lipid metabolism signal pathways. These findings provide novel insights into the mechanisms of TFs as a potent agent to treat acute liver injury in the future. However, further investigations are needed to elucidate in depth the mechanisms and clinical applications of this natural product.

## Figures and Tables

**Table 1:** Primer sequences used for real-time PCR.

Gene	GenBank	Full Name	Primer (5′-3′)
GAPDH	NM_008084.2	Glyceraldehyde-3-phosphatedehydrogenase	TGTGTCCGTCGTGGATCTGATTGCTGTTGAAGTCGCAGGAG
TNF-α	NM_013693.2	Tumour necrosis factor alpha	TATGGCCCAGACCCTCACAGGAGTAGACAAGGTACAACCCATC
IL-1β	NM_008361.3	Interleukin-1 beta	TCCAGGATGAGGACATGAGCACGAACGTCACACACCAGCAGGTTA
IL-6	NM_031168.1	Interleukin-6	CCACTTCACAAGTCGGAGGCTTACCAGTTTGGTAGCATCCATCATTTC
SREBP-1c	NM_011480.3	Sterol regulatory element-binding proteins-1c	CCGAGATGTGCGAACTGGAGAAGTCACTGTCTTGGTTGTTGATG
SCD1	NM_009127.4	Stearoyl-Coenzyme desaturase-1	ATGTCTGACCTGAAAGCCGAGAAGAGCACCAGAGTGTATCGCAAGAA

PCR, polymerase chain reaction; GAPDH, glyceraldehyde-3-phosphate dehydrogenase; TNF-α, tumor necrosis factor alpha; IL-1β, interleukin-1 β; IL-6, interleukin-6; SREBP-1c, sterol regulatory element-binding proteins-1c; SCD1, stearoyl-coenzyme A desaturase 1.

**Table 2:** Antibodies used for western blotting.

Antibody	Full Name	Source	Dilutions	Company
FXR	Farnesoid X Recepter	Rabbit	1:500	Bioss, Beijing, China
Nrf2	Nuclear erythroid factor 2-related factor 2	Rabbit	1:1000	Proteintech Group, Chicago, USA
Keap1	Kelch-like ECH-associated protein 1	Rabbit	1:1000	Proteintech Group, Chicago, USA
HO-1	Heme oxygenase-1	Rabbit	1:1000	Proteintech Group, Chicago, USA
NQO1	NAD(P)Hdehydrogenase(quinone 1)	Rabbit	1:1000	Proteintech Group, Chicago, USA
GCLC	Glutamate-cysteine ligase catalytic subunit	Rabbit	1:1000	Proteintech Group, Chicago, USA
GCLM	Glutamate-cysteineligase regulatory subunit	Rabbit	1:1000	Proteintech Group, Chicago, USA
FOXO3a	Forkhead box O3	Rabbit	1:1000	Proteintech Group, Chicago, USA
NF-κB	Nuclear factor kappa B	Rabbit	1:1000	Proteintech Group, Chicago, USA
COX-2	Cyclooxygenase-2	Rabbit	1:1000	Proteintech Group, Chicago, USA
HMGB1	High-Mobility Group Box 1	Rabbit	1:1000	Proteintech Group, Chicago, USA
ACC1	Acetyl-Coa carboxylase-1	Rabbit	1:1000	Proteintech Group, Chicago, USA
FASN	Fatty acid synthase	Rabbit	1:1000	Proteintech Group, Chicago, USA
CPT1	Carnitine palmitoyltransferase 1	Rabbit	1:1000	Proteintech Group, Chicago, USA
GAPDH	Glyceraldehyde-3-phosphatedehydrogenase	Rabbit	1:1000	Proteintech Group, Chicago, USA

## Abbreviations

ACC1	acetyl-CoA carboxylase-1
ALI	acute liver injury
ALT	alanine transferase
AST	aspartate transaminase
COX2	cyclooxygenase-2
CPT1	carnitine palmitoyltransferase 1
FASN	fatty acid synthase
FOXO3a	forkhead box O3
FXR	farnesoid X receptor
GAPDH	glyceraldehyde-3-phosphatedehydrogenase
GCLC	glutamate-cysteine ligasecatalytic subunit
GCLM	glutamate-cysteine ligase regulatory subunit
GSH-Px	glutathione peroxidase
HMGB-1	high-mobility group box 1
HO-1	heme oxygenase-1
IL-1β	interleukin-1 β
Keap1	Kelch-likeECH-associated protein 1
MDA	malondialdehyde
NF-κB	nuclear factor-kappa B
Nrf2	nuclear erythroid factor2-related factor 2
NQO1	NAD(P)H dehydrogenase(quinone 1)
SCD1	stearoyl-coenzyme A desaturase 1
SOD	superoxide dismutase
SREBP-1c	sterol regulatory element-binding proteins-1c
TC	total cholesterol
TFs	total flavonoids from *Rosa laevigata* Michx fruit
TG	total triglyceride
TNF-α	tumor necrosis factor alpha
